# A global dataset of onshore wind turbines with site-specific historical (1989–2018) and future (2030–2059) wind resources across 89 countries

**DOI:** 10.1038/s41597-026-07290-4

**Published:** 2026-04-21

**Authors:** Christopher Jung, Dirk Schindler

**Affiliations:** Environmental Meteorology, University of Freiburg, Werthmannstrasse 10, D-79085 Freiburg, Germany

## Abstract

The expansion of wind energy is a key strategy for mitigating global climate change. To support this goal, consistent global-scale datasets of existing wind turbines are essential for planning the future deployment of wind energy. Here, we introduce GOWIRES, a comprehensive global dataset of onshore wind turbines. GOWIRES provides detailed information on 416,417 horizontal-axis wind turbines (HAWT) across 89 countries. The dataset includes geographic coordinates, key technical specifications, and site-specific environmental characteristics for each wind turbine. In addition, GOWIRES provides historical (1989–2018) and future (2030–2059) site-specific wind resource data. Wind resources are characterized by mean wind speed, mean wind power density, Weibull parameters, power law exponents, and air density. Future Weibull parameters are based on simulations from 13 statistically downscaled global climate models under the SSP2-4.5 and SSP5-8.5 scenarios. GOWIRES is a valuable resource for energy and climate research, as well as for applications in wind energy development, grid and infrastructure planning, and policy-making.

## Background & Summary

Wind energy, as a climate-friendly power source, continues to gain momentum worldwide. By 2024, global installed wind capacity had reached 1.13 TW^1^, with onshore installations accounting for 93% of the total. Notably, 2024 marked the first time that land-based wind capacity exceeded 1 TW^1^.

The ongoing necessity to reduce greenhouse gas emissions to mitigate climate change is driving further expansion of wind energy worldwide^2^. At the same time, existing installations typically require replacement after 20–25 years of operation through a process known as repowering^3^. The conversion, construction, and dismantling of wind turbines leads to continuously evolving wind turbine fleets^4^.

In terms of technology, horizontal axis wind turbines (HAWTs) remain the dominant type worldwide. In contrast, vertical axis wind turbines are used primarily for small-scale and niche applications^5^. Key characteristics of wind turbines include rated power (*PR*), rotor diameter (*RD*), and hub height (*HHUB*)^6^. Unlike conventional power plants, wind turbines are highly dependent on atmospheric conditions, which can vary considerably over time and space^7^. Furthermore, the wind resource itself is subject to climate change-related fluctuations^8^.

Thus, comprehensive and detailed datasets on wind turbine fleets are essential across many fields. Such datasets serve as an increasingly important foundation for addressing questions related to wind energy, climate change, and infrastructure, from the global to the local scale. They enable researchers, policymakers, businesses, and civil society to make data-driven decisions and gain new insights.

There are various sources of wind turbine data. For some countries, such as Canada^9^, France^10^, Germany^11^, Sweden^12^, and the United States^13^, freely accessible datasets exist that typically provide precise coordinates and key technical specifications of wind turbines. OpenStreetMap (OSM) is a crucial global source for onshore wind turbines^14^, offering exact coordinates worldwide and, for some sites, additional technical information. Furthermore, wind turbine coordinate datasets based on satellite imagery are available for South Africa^15^ and China^16^.

However, information on existing and future wind resources, which is essential for calculating wind energy yields, is generally absent from national datasets, OSM, and satellite-derived datasets. A recent dataset fills this gap by including future wind power predictions at the individual wind turbine scale but is limited to Germany^17^.

These examples highlight the need for a comprehensive and detailed global scale wind turbine dataset that not only covers precise coordinates, technical characteristics, and information regarding wind turbine’s environment but also provides historical and future wind resource information.

GOWIRES^18^ addresses the lack of global, standardized wind turbine datasets by providing detailed information on 416,417 wind turbines across 89 countries. Unlike many previous datasets that were limited to location coordinates, GOWIRES offers a wide range of additional attributes critical for power output calculation in recent and future periods. All attributes are summarized in Table 1.

**Table 1:** Variables contained in GOWIRES^18^, including their parent category and abbreviation.

parent category	description	abbreviation
basic	full id	full_id
basic	longitude	longitude
basic	latitude	latitude
basic	country	country
basic	source	source
environment	elevation	elevation
environment	slope	slope
environment	aspect	aspect
environment	forest area (binary indicator)	forest
environment	urban area (binary indicator)	urban
turbine properties	rated power	power
turbine properties	rotor diameter	diameter
turbine properties	specific rating	specific_rating
turbine properties	hub height	hub_height
turbine properties	manufacturer	manufacturer
historical wind resource	mean wind speed in 1989–2018	WSM
historical wind resource	mean wind power density in 1989–2018	WPD
historical wind resource	air density in 1989–2018	AD
historical wind resource	power law exponent in 1989–2018	PLE
historical wind resource	Weibull scale parameter in 1989–2018	weibull_c_hist
historical wind resource	Weibull shape parameter in 1989–2018	weibull_k_hist
future wind resource	Weibull scale parameter in 2030–2059 (26 model–scenario combinations)	weibull_c_SSP
future wind resource	Weibull shape parameter in 2030–2059 (26 model–scenario combinations)	weibull_k_SSP
future wind resource	projection plausibility flag	plausibility_flag

The dataset is structured into five main categories. The “basic” category contains coordinates and country information, with wind turbine coordinates sourced primarily from the OSM dataset. The accuracy and completeness of these coordinates were validated against national wind turbine inventories.

The “environment” category provides information on elevation, aspect, and slope, and indicates whether a wind turbine is situated in forest area, urban area, or neither. This context is crucial for planning processes.

The “turbine properties” category includes technical details such as *PR*, *RD*, specific rating (*SR*), *HHUB*, and manufacturer. These data were compiled by merging information from national wind turbine registries with OSM data. The data enables the identification of large-scale variations in wind turbine characteristics.

The “historical wind resource” category encompasses key variables describing wind resource conditions in 100 m for 1989–2018: mean wind speed (*WSM*), mean wind power density (*WPD*), air density (*AD*), power law exponent (*PLE*), and Weibull scale (*c*) and shape (*k*) parameters. These data facilitate the calculation of wind speed distributions, extrapolation to different *HHUB*, and adjustments for air density when estimating energy yield.

Wind resources can vary significantly on a local scale due to differences in land use and exposure. To adequately capture the high spatial variability of wind resources, data in this category were derived and calculated from the high-resolution global wind speed model “GloWiSMo”^19^. In addition to prior validation with 598 globally distributed wind speed measurements in 10 m, GloWiSMo was further verified using wind speed data at *HHUB* from 79 wind turbines.

Another important feature is that the fifth category, “future wind resource,” provides Weibull parameters for the period 2030–2059. GOWIRES includes 26 variants of Weibull parameters to capture the uncertainties associated with future wind resource development. These variants result from the combination of 13 global climate models and the two Shared Socioeconomic Pathways, SSP2-4.5 and SSP5-8.5. Because the global climate models are available at a coarse spatial resolution, the Weibull parameters were statistically downscaled to the wind turbine scale using the quantile mapping method and GloWiSMo^20^.

**Fig. 1:**
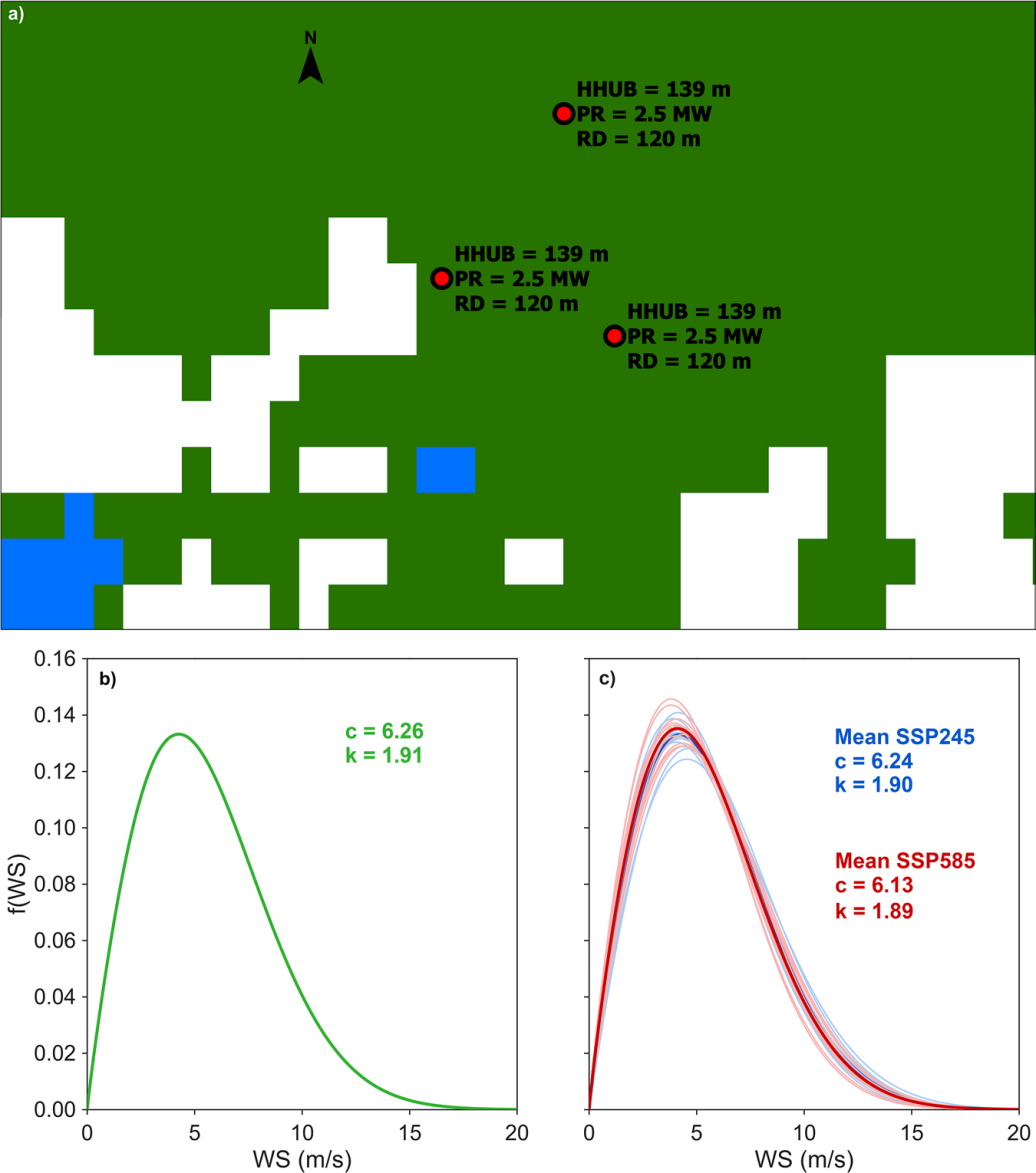
Features of an illustrative wind farm: a) wind turbine locations shown as red points, with hub height (*HHUB*), rated power (*PR*) and rotor diameter (*RD*) overlaid on land cover (green, forest; blue, urban); b) historical Weibull probability density function of the southernmost wind turbine; and c) future Weibull probability densities under SSP2-4.5 (blue curves) and SSP5-8.5 (red curves) of the southernmost wind turbine by 13 global climate models.

Figure 1 summarizes the main components of GOWIRES at the scale of an exemplary wind farm site. Subplot a) overlays the wind turbine coordinates with land-cover information and the technical attributes stored in GOWIRES. The subplots b) and c) show the Weibull probability density functions for one wind turbine of the same wind farm under historical conditions and under the CMIP6 scenarios SSP2-4.5 in blue and SSP5-8.5 in red.

The breadth and depth of GOWIRES enable a wide range of applications across various sectors. For example, in energy and climate research, the dataset can be used to analyze future wind energy yields considering the uncertainties of climate change. The wind energy sector can utilize GOWIRES for repowering assessments and site selection. Moreover, the dataset serves as a solid foundation for large-scale grid and infrastructure planning. Policymakers can draw on GOWIRES to evaluate whether wind energy expansion aligns with established targets. These examples represent only a fraction of the potential applications made possible by GOWIRES. Given the continual changes in the global wind turbine fleet, GOWIRES will be updated annually.

## Methods

The construction of the GOWIRES dataset followed five principal stages. First, we extracted and filtered up-to-date (July 2025), high-precision wind turbine coordinates from OSM and national inventories. Second, we assigned the technical specifications of the wind turbines by combining national wind-turbine inventories with OSM attributes. Third, we characterized the surrounding environment of each site using land-use and terrain models. Fourth, we derived the site-specific wind resource for the historical period 1989–2018 by means of GloWiSMo. Fifth, we applied global climate model data and the quantile mapping method to project future wind resources (2030–2059) under two SSP scenarios.

### Wind turbine sites and properties

Wind turbine sites were extracted from OSM using QGIS 3.38 and the QuickOSM plugin. The data were collected in July 2025. The query targeted the key–value pair *generator:source = wind* and *plant:source = wind*. It was executed separately for every country hosting onshore wind power in 2024 according to the International Renewable Energy Agency^1^. To stay within Overpass Application Programming Interface (API) limits, spatially extensive countries (Canada, the United States, and Russia) were processed at subnational scale. The resulting shapefiles were merged into a global layer and the attribute fields were harmonized.

Completeness and accuracy of this global OSM dataset was evaluated against national wind-turbine inventories from Canada^9^, France^10^, Germany^11^, Sweden^12^, and the United States^13^. Details are provided in the Technical Validation. Turbine locations present in these reference datasets but absent from OSM were appended, producing an extended global layer.

To restrict the dataset to conventional HAWT, entries tagged *type = vertical_axis* were identified and filtered. To verify the OSM classification of vertical-axis turbines, the World Imagery basemap was overlaid in ArcGIS Pro^21^ and visually checked. Elements containing texts such as “solar” or “heat_pump” were also filtered^15^.

Both OSM and the national inventories provide records of *RD*, *HHUB*, *PR*, and turbine manufacturer for many wind turbines. After harmonizing coordinates, attribute tables were cross-matched using turbine location as the primary key. In cases where both OSM and national inventories provided entries for *RD*, *HHUB*, *PR*, or turbine manufacturer, the following deterministic rules were applied:

(i) National registry values were systematically prioritized over OSM entries, as their overall completeness is substantially higher.
(ii) If no national value was available, the corresponding OSM value was retained.
(iii) Implausible technical values were removed. Specifically, *HHUB* values below 10 m or above 200 m, *PR* values below 0.1 MW or above 15 MW, and *RD* values below 20 m or above 250 m were considered physically implausible for onshore horizontal-axis wind turbines and were therefore excluded.

The completeness of turbine property attributes is 23.2% for manufacturer, 31.7% for *HHUB*, 35.5% for *RD*, and 42.8% for *PR*.

Whenever both *RD* and *PR* were available, the specific rating (*SR*) was computed to enrich the technical data:

1 $${SR}=\frac{{PR}}{\pi {\left(\frac{{RD}}{2}\right)}^{2}}$$

To identify duplicates in the wind turbine dataset, each turbine’s nearest neighbor was determined in ArcGIS Pro via the “Near” tool, yielding the nearest wind turbine’s ID and the nearest distance (*ND*). These pairwise relations were processed in MATLAB 2025a. Pairs with *ND* < 15 m were flagged as duplicates, and the second record in the pair was marked for removal. Additionally, any pair with known *RD* values was checked for physical overlap. If

2 $${ND}\le 0.5\cdot {\min }({RD})$$

the wind turbine with the smaller *RD* was flagged as a duplicate. The completeness of *SR* is 35.2%.

**Fig. 2:**
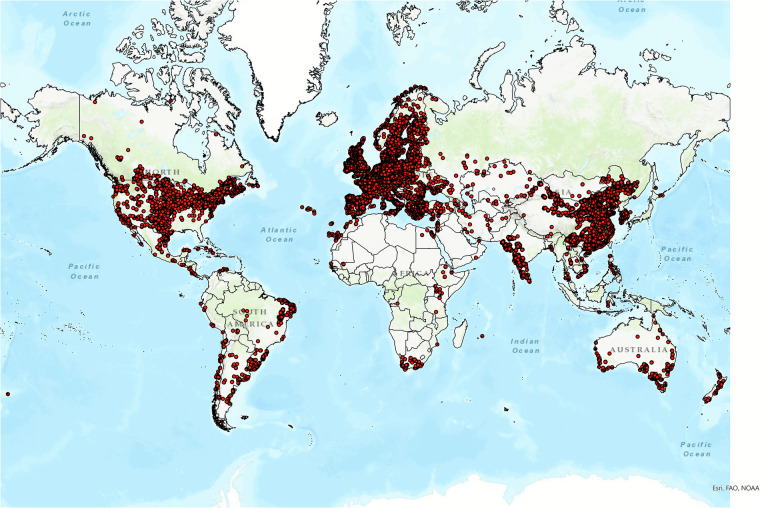
Global distribution of the 416,417 wind turbine sites included in GOWIRES.

The 416,417 final wind turbine sites included in GOWIRES are shown in Fig. 2.

### Environment

Basic site characteristics were assigned to every wind turbine location. Elevation was taken from a global 250 m × 250 m digital elevation grid^22^. In addition, terrain slope and aspect grids were computed from the same digital elevation grid via the ArcGIS Pro tools “Slope” and “Aspect”.

Land cover, specifying whether each wind turbine lies within forest or urban fabric, was derived from the Copernicus Global Land Service: Land Cover 100 m product^23^. All raster datasets were sampled at the wind turbine coordinates using the ArcGIS Pro tool “Extract Multi Values to Points”.

### Historical wind resource

The GloWiSMo model^19^ was used to estimate the wind resource specific to wind turbine sites during the historical period 1989–2018. GloWiSMo provides near-surface wind-speed L-moments in 10 m above ground level (agl) and power law exponents (*PLE*) on a 250 m × 250 m grid estimated based on 1) long-term, globally distributed, hourly wind speed measurements, 2) ERA5 reanalysis data, and 3) eleven terrain and land-cover-related predictors. Based on the predicted L-moments, it derives Kappa distribution parameters^24^ and its *PLE* values enable to extrapolate the wind speed distributions to any *HHUB*^25^. GloWiSMo’s ability to estimate wind resources at *HHUB* is demonstrated in the Technical Validation.

For development of GOWIRES, the site-specific wind-speed distributions in 10 m agl provided by GloWiSMo were transformed from their native Kappa distribution representation into Weibull form, as the Weibull distribution is more widely used in wind-energy analyses^26^, and thus easier to integrate into subsequent applications^27^.

For each wind turbine site, the GloWiSMo Kappa parameters served to generate random wind speed *(*${{WS}}_{10m}$*)* samples for a height of 10 m. A synthetic dataset equivalent to 30 years of hourly records was produced to satisfy standard climatological conventions.

The quantile function (*F*) of the Kappa distribution is calculated as^28^:

3 $${{WS}}_{10m}\left(F\right)=\varepsilon +\frac{\alpha }{\kappa }\left[1-{\left(\frac{1-{F}^{h}}{h}\right)}^{\kappa }\right]$$

with *ε* the location parameter, *α* the scale parameter, and the both shape parameters *h* and *κ*.

Next, Weibull distributions were fitted to the derived *WS* samples in MATLAB using maximum-likelihood estimation, yielding the shape (${k}_{10m}$) and scale (${c}_{10}$) parameters in 10 m.

The Weibull probability density function (*f*) is defined as^29^:

4 $$f\left({{WS}}_{10m}\right)=\frac{{k}_{10m}}{{c}_{10m}}{\left(\frac{{{WS}}_{10m}}{{c}_{10m}}\right)}^{{k}_{10m}-1}\exp \left[-{\left(\frac{{{WS}}_{10m}}{{c}_{10m}}\right)}^{{k}_{10m}}\right]$$

The wind speed distributions at 10 m agl are insufficient for wind energy applications. Thus, the distribution parameters were extrapolated to *HHUB* = 100 m following the widely used Justus and Mikhail method^30–32^. The following equations were applied for calculating *k* at hub height (${k}_{{HHUB}}$) and *c* at hub height (${c}_{{HHUB}}$):

5 $${k}_{{HHUB}}={k}_{10m}\cdot \left[\frac{1-0.0881\cdot \mathrm{ln}\left(\frac{10m}{10}\right)}{1-0.0881\cdot \mathrm{ln}\left(\frac{{HHUB}}{10}\right)}\right]$$

and

6 $${c}_{{HHUB}}={c}_{10m}{\left(\frac{{HHUB}}{10m}\right)}^{{PLE}}$$

The Weibull parameters ${c}_{{HHUB}}$ and ${k}_{{HHUB}}$ allows calculating two core wind-resource metrics: the mean wind power density (*WPD*) and the mean wind speed (*WSM*). Both quantities were computed at every wind turbine site and added to the GOWIRES dataset.

*WPD* was calculated by^31,32^:

7 $${WPD}=\frac{1}{2}\rho \,{{c}_{{HHUB}}}^{3}\varGamma \left(1+\frac{3}{{k}_{{HHUB}}}\right)$$

with $\rho$ the air density being available from^33^ and $\varGamma$ the Gamma function.

The following equation was applied for determining *WSM*^31,32^:

8 $${WSM}={c}_{{HHUB}}\cdot \varGamma \left(1+\frac{1}{{k}_{{HHUB}}}\right)$$

### Future wind resource

Future wind resources are represented in GOWIRES by 13 CMIP6 global climate models: ACCESS-CM2^34^, ACCESS-ESM1-5^35^, CanESM5^36^, CESM2-WACCM^37^, CMCC-CM2-SR5^38^, CMCC-ESM2^39^, EC-Earth3^40^, IITM-ESM^41^, IPSL-CM6A-LR^42^, MPI-ESM1-2-HR^43^, MPI-ESM1-2-LR^44^, MRI-ESM2-0^45^, and NorESM2-MM^46^.

The wind speed distributions of the global climate models were statistically downscaled to the wind turbine scale via quantile mapping^20,47^. The steps involved in quantile mapping were identical for all global climate models. First, 10 m near-surface wind speed time series from the CMIP6 models were extracted at every wind turbine location for the historical period and for the 2030–2059 horizon under the SSP2-4.5 and SSP5-8.5 scenarios. Then, empirical quantile functions were subsequently computed for both periods. Next, the historical model quantile function was matched with the future model quantiles to derive cumulative probabilities for each projected value. These probabilities were then transferred onto the GloWiSMo-derived wind speed values by evaluating the inverse of the historical Weibull fit. The synthetic wind speed values were re-fitted to a Weibull distribution using maximum-likelihood estimation, yielding site-specific future *c*_10*m*_ and *k*_10*m*_. Finally, the downscaled Weibull parameters were extrapolated to *HHUB* using the Justus and Mikhail method^30^ as explained for the historical period.

In summary, the downscaling procedure transfers projected large-scale distributional changes from CMIP6 models to the turbine scale while preserving the spatial structure of the historical GloWiSMo climatology. The resulting datasets therefore represent statistically downscaled GCM-based projections and do not constitute dynamically downscaled regional climate simulations. Consequently, the projections reflect climate-model-driven shifts in free-stream wind statistics rather than physically resolved mesoscale flow dynamics.

## Data Records

GOWIRES^18^ is publicly available from Zenodo (10.5281/zenodo.18768952). The dataset can be downloaded in Excel (xlsx), Shapefile (shp), GeoPackage (gpkg), and comma-separated values (CSV) formats. Shapefile and GeoPackage are suitable for use with GIS software, while Excel and CSV formats are compatible with spreadsheet programs. The data content is identical across all formats. The coordinates are provided in the WGS84 (EPSG:4326) coordinate system. The total file sizes are 180 MB (xlsx), 1.17 GB (shp), 302 MB (gpkg), and 149 MB (CSV). Each file contains 416,417 rows representing wind turbines distributed worldwide. For each wind turbine, there are 74 columns organized into five main categories:

- “Basic,” containing five columns: the turbine ID, longitude, latitude, country, and coordinate source information.
- “Environment,” comprising elevation, slope, aspect, forest area, and urban area.
- “Turbine properties,” providing information on rated power, rotor diameter, specific rating, hub height, and manufacturer.
- “Historical wind resource,” including mean wind speed, mean wind power density, air density, power law exponent, and Weibull parameters.
- “Future wind resource”, containing 52 columns representing Weibull parameters projected using 13 global climate models for the SSP2-4.5 and SSP5-8.5 scenarios and a column for projection plausibility.

Column headings are labeled with descriptive abbreviations. A comprehensive overview of all variables, their units, and abbreviations is provided in the “Metadata” Excel file included with the dataset. GOWIRES will be updated on yearly basis to account for changes in the global wind turbine fleet.

## Technical Validation

Two key aspects are evaluated as part of validation. First, site validation assesses the accuracy and completeness of the wind turbine locations. Second, wind resource validation examines how accurately the wind resource is characterized at the wind turbine sites.

### Site validation

To assess the coordinate accuracy and completeness of OSM wind turbine sites, five countries with authoritative national inventories (Canada^9^, France^10^, Germany^11^, Sweden^12^, USA^13^) were examined. These countries account for a total of 26.1% of the world’s installed onshore capacity in 2024^1^. For each country, the OSM coordinates and the national registry entries were projected into a common Coordinate Reference System and pairwise nearest neighbors were identified with the ArcGIS Pro “Near” tool. This yielded the minimum cross-dataset distance for every wind turbine. The results were classified into four categories: (i) category “Match” including wind turbines present in both sources (distance below 100 m), (ii) category “National exclusive” including wind turbines present only in the national inventory (no OSM match within 500 m), (iii) category “OSM exclusive” including wind turbines present only in OSM (no national inventory match within 500 m), and (iv) the category “Short distance” refer to wind turbines matched between 100 m and 500 m apart.

**Fig. 3:**
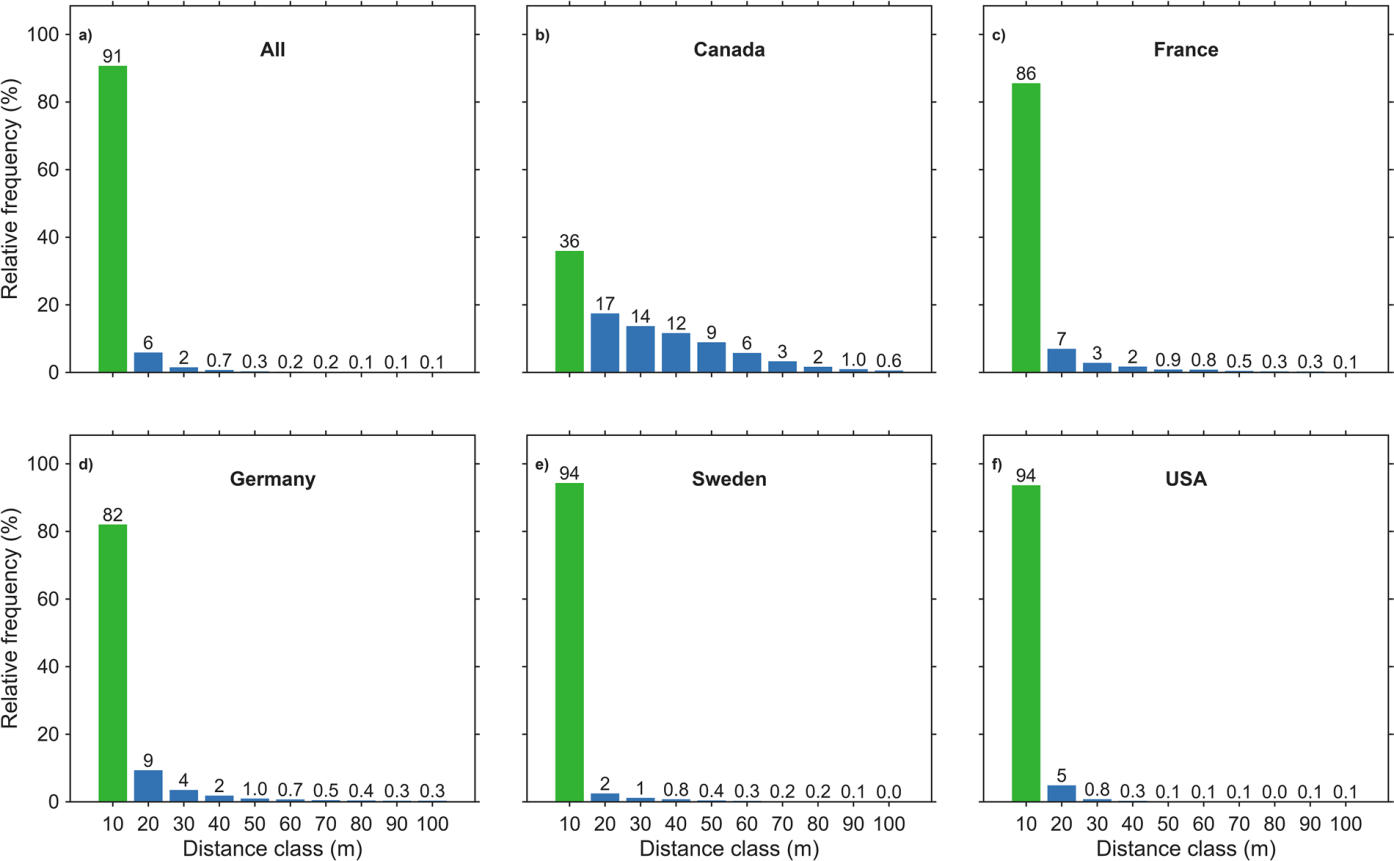
Histograms of the distances between OpenStreetMap (OSM) wind turbine sites and national inventories wind turbine sites for the wind turbines present in both sources (distance < 100 m) for (**a**) all five countries, (**b**) Canada, (**c**) France, (**d**) Germany, (**e**) Sweden, and (**f**) the USA.

The accuracy of wind turbines in the category “Match” is presented in Fig. 3 by histograms of the nearest-neighbor distances (10 m bins) between wind turbines recorded in OSM and in the national inventories. Across all five countries (Figs. 3a), 90.7% of matched wind turbines lie within 10 m of each other; a further 5.9% fall in the 11–20 m class and 1.5% in the 21–30 m class, while every higher bin accounts for well under 1.0% of wind turbine sites.

The country-level distributions differ. The largest discrepancies occur in Canada (Fig. 3b), where only 36.0% of wind turbines fall below 11 m, although large mismatches remain uncommon: distances >50 m together represent just 12.3%. France (Fig. 3c) and Germany (Fig. 3d) likewise exhibit high positional agreement, with more than 80% of wind turbines matched to within 10 m. By contrast, Sweden (Fig. 3e) and the United States (Fig. 3f) show the highest consistency, with 94.3% and 93.7% of wind turbines within 10 m and all categories above 40 m contributing less than 1%.

Considering these results and accounting for the several-meter tower diameter typical of HAWT underscores that the positional accuracy of the OSM data is generally very high.

**Fig. 4:**
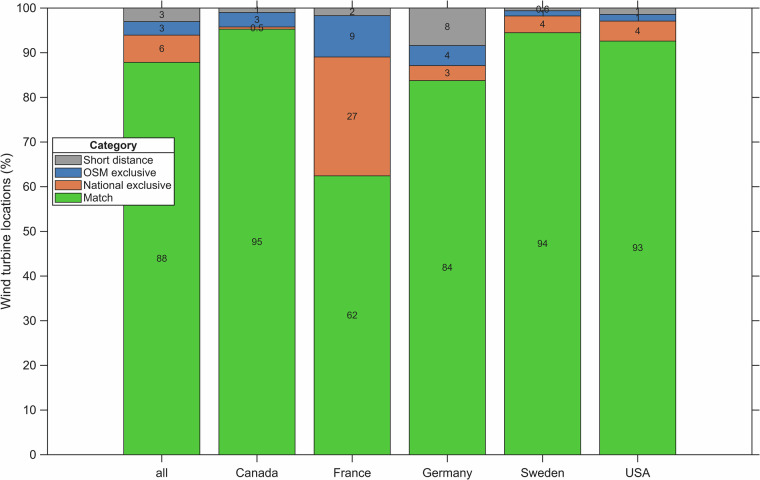
Percentage of agreement of OpenStreetMap (OSM) wind turbine sites and national inventory in Canada, France, Germany, Sweden, the USA, and all five countries including the categories “Match”, “National exclusive”, “OSM exclusive”, and “Short distance”.

Figure 4 summarizes the percentage share of wind turbine matches, OSM-exclusive sites, national-exclusive sites, and short distance matches. For all countries, matches account for 87.8% of all locations, indicating that OSM provides high coverage relative to the authoritative national registers. 6.1% of sites appear only in national inventories, and 3.0% only in OSM data. A further 3.0% remain ambiguous (“Short distance” sites) because their nearest-neighbor distances lie between 100 m and 500 m. For these distances, the duplicate-check procedure detailed in the methodology is relevant.

Country-level differences are also notable. In Canada, Sweden and the United States, matched wind turbines account for 92.6–95.3% of all sites, whereas in France the match rate is only 62.4%, with large fractions appearing exclusively in OSM or in the national register. Germany stands out with 8.4% of sites falling into the “Short distance” category; this likely reflects the very high density of wind turbines there, which makes close but independent neighbors more common.

The presence of both OSM-exclusive and national-exclusive sites can be traced in part to imperfect temporal overlap: even if both inventories are downloaded on the same day, their underlying surveys are rarely synchronized, so wind turbines under construction or recently retired may appear in only one register. The small national-exclusive fraction likely reflects wind turbines awaiting upload to OSM or decommissioned turbines that remain in official datasets, whereas the OSM-exclusive share showcases the value of community mapping, which often captures new installations or upgrades sooner than national reporting systems. Some OSM-exclusive entries also correspond to small turbines or vertical-axis machines, which are absent from national inventories and removed during GOWIRES’s filtering step. Collectively, these factors highlight the need for regular updates to GOWIRES and for rigorous duplicate and filtering procedures as explained and applied in the Methods section.

### Wind resource validation

The validation of GloWiSMo is critical because it forms the basis for the wind resources included in GOWIRES. GloWiSMo has already undergone extensive validation at 10 m agl by comparison with national meteorological measurements^19^. In a rigorous global test against 598 wind speed time series, the model reproduced observed *WSM* with a coefficient of determination (*R*^*2*^) of 0.83 and a mean absolute error (*MAE*) of 0.53 m/s.

In addition, here, we also evaluated GloWiSMo against operational wind speed measurements at 79 wind turbines in eight German wind farms. Multi-year hourly wind speed records at hub height were supplied under confidentiality by the operator “Das Grüne Emissionshaus” (Freiburg im Breisgau, Germany). This measured wind speed data had already been used in an earlier study to validate wind speed models^48^. For each wind turbine, the GloWiSMo wind speed time series (extrapolated to the site-specific *HHUB*) were compared with the measured data over the common period. The analysis compared (i) the long-term *WSM* values and (ii) the deciles of the wind-speed distribution (10% (*D*_*10*_), 20% (*D*_*20*_), …, 90% (*D*_*90*_)). These metrics provide a detailed view of the model’s performance across the entire wind speed range relevant to wind turbine operation.

Table 2 summarizes the validation metrics *MAE*, root mean squared error (*RMSE*) and mean absolute percentage (*MAPE*). The GloWiSMo-derived wind-speed distributions capture the average wind speed conditions very well: *MAE* for *WSM* is 0.38 m/s, and *MAPE* is only 7.2%. The model performs especially well at the median decile (*D*₅₀), where *MAE*, *RMSE*, and *MAPE* reach their minima of 0.30 m/s, 0.38 m/s, and 5.7%. However, the model performance deteriorates toward both tails of the distribution: *MAPE* values rise to 24.9% at *D*_*10*_ and to 20.9% at *D*_*90*_.

**Table 2:** Validation of historical GloWiSMo wind speed distributions against observed wind speed measurements at 79 wind turbine sites for mean wind speed *(WSM*) and wind speed deciles (*D*).

variable	*MAE* (m/s)	*RMSE* (m/s)	*MAPE* (%)
*WSM*	0.38	0.47	7.2
*D*_*10*_	0.66	0.79	24.9
*D*_*20*_	0.59	0.71	17.4
*D*_*30*_	0.50	0.61	12.3
*D*_*40*_	0.39	0.48	8.2
*D*_*50*_	0.30	0.38	5.7
*D*_*60*_	0.37	0.45	6.4
*D*_*70*_	0.65	0.72	10.0
*D*_*80*_	1.17	1.23	16.1
*D*_*90*_	1.77	1.87	20.8

Performance metrics are mean absolute error (*MAE*), root mean squared error (*RMSE*), and mean absolute percentage error (*MAPE*).

**Fig. 5:**
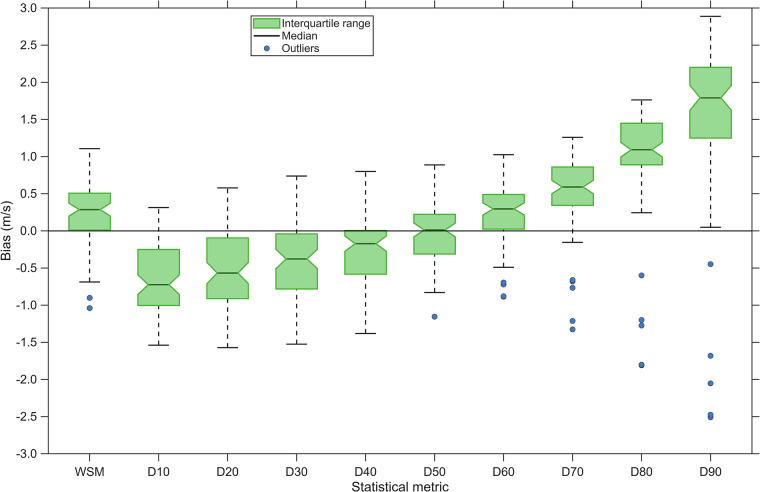
Boxplots of the errors (Bias) of mean wind speed (*WSM*) and the deciles of the wind speed distribution (*D*_10_, *D*_20_, …, *D*_90_) including 79 wind turbines in eight German wind farms.

Figure 5 shows the biases of *WSM* and the deciles for all validated wind turbine sites as boxplots. *WSM* is only slightly overestimated on average, with a median bias of 0.29 m/s. The median decile derived from GloWiSMo shows no bias toward the measured values. The most relevant biases occur at the edges of the distribution. The low percentiles tend to be underestimated and the higher ones overestimated.

The observed biases can mainly be attributed to wake effects within wind farms. Because wakes reduce the measured wind speed, they lead to a slight positive bias when modeled values (which are free of wake losses) are compared with in-situ measurements^49^. This mechanism also explains the more pronounced deviations at the upper tail of the distribution: as wind speed increases, wake deficits become stronger, so the observations fall increasingly below the free-stream conditions assumed by the model. Conversely, the lower tail of the distribution is less affected, though a slight negative bias remains where wake interaction tends to vanish.

Once wake-induced wind speed deficits are acknowledged^49^, the small residual errors, as well as *MAE*, *RMSE*, and *MAPE*, shrink further, demonstrating that GloWiSMo reliably reproduces the free-stream wind speed climate. In short, the model fulfils its intended purpose. It delivers an accurate representation of undisturbed flow conditions at the wind turbine sites.

For a complete validation of the selected CMIP6 models, which form the basis for future estimates, please refer to^4^.

It should be noted that a very small fraction of wind turbine sites (≈0.1%) exhibits extreme projected changes in mean wind speed for individual CMIP6 models (MPI-ESM1-2-LR under SSP2-4.5, MRI-ESM2-0 under SSP2-4.5, and CESM2-WACCM under SSP5-8.5). While these values are unlikely to represent robust climate signals, they arise from individual ensemble members under specific large-scale circulation patterns and from the amplification inherent in statistical downscaling. These extreme projections are therefore retained and flagged in the dataset to preserve the complete ensemble information and to ensure transparency and reproducibility.

## Usage Notes

The 2026 release of GOWIRES (GOWIRES_V1) represents a snapshot of global onshore wind turbines and wind resources with data as of July 2025. Future versions of GOWIRES are planned on an annual cadence because wind power is expanding rapidly and OSM/national registers are periodically updated. Subsequent releases of GOWIRES will also extend the historical (1989–2018) GloWiSMo baseline to more recent periods. Users who require the most recent fleet configuration should check the repository for newer releases.

Despite extensive cross-checking, neither completeness nor perfect positional accuracy can be guaranteed. Some wind turbines may be missing or slightly misplaced, especially in regions with sparse reporting.

GOWIRES is a global wind infrastructure and resource dataset, not a complete engineering dataset of turbine specifications. Technical attributes such as hub height and rated power are not available for all wind turbines, particularly in regions where national registries do not provide this information. Users conducting analyses that require these parameters (e.g. energy yield estimation) should either restrict their study to wind turbines with complete technical records or apply appropriate imputation methods. While reasonable care has been taken to harmonize manufacturer and turbine information, no warranty is given regarding the completeness or absolute accuracy of individual entries.

For records originating from OSM, the field “full id” corresponds to the original OSM feature identifier and thus enables direct interoperability with OSM-based datasets. For entries derived from national inventories, “full id” represents a GOWIRES-specific identifier.

The Weibull parameters in GOWIRES (both historical and future) represent free-stream wind speed distributions in 100 m above ground level. Users analyzing wind farms should apply an appropriate wake loss model to account for the actual inflow conditions.

The future Weibull parameters are derived from CMIP6 global climate model projections and therefore carry inherent uncertainties. Rare, extreme changes in projected mean wind speed occur at fewer than 0.1% of wind turbine sites for some model–scenario combinations (MPI-ESM1-2-LR under SSP2-4.5, MRI-ESM2-0 under SSP2-4.5, and CESM2-WACCM under SSP5-8.5). Such deviations are unlikely to represent robust climate signals and instead likely reflect model-specific artefacts or local amplification effects. These rare extreme changes in projected mean wind speed (> ± 5 m/s) are flagged in the dataset in the column “projection plausibility flag”. Users are therefore encouraged to interpret future wind resource changes using ensemble statistics (means, medians, or interquartile ranges). Where site-specific robustness is required, users may apply their own plausibility thresholds or outlier screening tailored to the application.

GOWIRES may serve as input for machine-learning applications. However, users should account for spatial autocorrelation when designing training and validation schemes (e.g. via spatial or block cross-validation) to avoid information leakage. In addition, attribute completeness varies across variables; therefore, appropriate filtering, imputation, or feature selection strategies should be applied.

When redistributing the data, please observe the license requirements of the original sources. Contributions and error reports are welcome by email to the authors.

## Data Availability

GOWIRES is publicly available via Zenodo at 10.5281/zenodo.18768952. The dataset can be downloaded in Excel (xlsx), comma-separated values (CSV), Shapefile (shp), and GeoPackage (gpkg) formats.

## Code availability

A MATLAB script for applying GOWIRES wind resource data is publicly available and can be downloaded from the repository accompanying the data descriptor (10.5281/zenodo.18768952). It reads the data, allows site and scenario selection, and computes the Weibull probability density function, mean wind speed, and mean wind power density as described.
